# Cross-sectional study of physical activity, dietary habits, and mental health of veterinary students after lifting of COVID-19 pandemic measures

**DOI:** 10.1371/journal.pone.0291590

**Published:** 2023-09-14

**Authors:** Daniela Luethy, Traci M. Krueger, Erica Cuneo, Julia R. Varnes, Jorge A. Hernandez

**Affiliations:** 1 Department of Large Animal Clinical Sciences, University of Florida College of Veterinary Medicine, Gainesville, Florida, United States of America; 2 College of Public Health and Health Professions, University of Florida, Gainesville, Florida, United States of America; 3 College of Veterinary Medicine, University of Florida, Gainesville, Florida, United States of America; Lahore Medical and Dental College, PAKISTAN

## Abstract

Mental illness is an important public health concern in veterinary students. Recent literature has demonstrated a negative effect of the COVID-19 pandemic on veterinary students’ mental health. Little literature to date has evaluated the mental health of veterinary students affected by the COVID-19 pandemic after most pandemic measures have been lifted. The objective of this study was to describe physical activity, diet, and mental health in veterinary students after pandemic measures were lifted. A secondary objective was to examine the association between depression symptoms and exposure factors in this cohort of veterinary students. In a cross-sectional study, veterinary students (n = 487) at a public university received an online survey with questions regarding their physical activity, diet, stress, and self-rated symptoms across 11 mental health domains. Logistic regression was used to quantify the association between exposure factors and depression symptoms. One-hundred and twelve students completed the survey. Sixty-three (56%) respondents met the criteria for concern within the mental health domain of depression, 75 (67%) for anxiety, and 16 (14%) for suicidal ideation. Fourth year students had the lowest weekly vigorous physical activity (median 0.5 hours). The odds of self-reported depression symptoms were 8 times lower in students engaged in high levels of vigorous exercise compared to students engaged in low levels, after controlling for number of years in the program (p = 0.02). Mental health concerns were high in this group of veterinary students.

## Introduction

Mental illness is a growing public health concern in veterinarians and veterinary students and has become an important point of discussion within the veterinary profession. Recent literature has demonstrated that the proportional mortality ratio for suicide in both male and female veterinarians is higher than that of the general US population [1]. In a survey study, younger veterinarians were found to be at highest risk of serious psychological distress, with high student debt and low income identified as contributing factors [2]. A more recent study also showed that the mental health of veterinarians declined over the past few years, likely driven by the COVID-19 pandemic as well as labor shortages [3]. Recent literature has similarly demonstrated an effect of e-learning during the COVID-19 pandemic on veterinary students’ mental health, with high levels of anxiety reported in veterinary students during the COVID-19 pandemic [4]. Excessive workload, concerns about academic performance, student debt, personal relationships, and the competitive atmosphere associated with professional education have all been identified as factors that contribute to psychological distress in veterinary students prior to the COVID-19 pandemic [5–8]. During the COVID-19 pandemic, difficulty sleeping, stress associated with confinement, and family conflicts were identified as risk factors for anxiety among veterinary students [4].

Poor physical health has been identified as a predictor of poor mental health in veterinary students [6, 9]. A recent study found that veterinary students who had a higher number of daily meals, more days of exercise, and more frequent contact with support systems had higher psychological well-being scores, but this data was collected in 2019 prior to the COVID-19 pandemic [9]. Physical activity was reduced among college students during the COVID-19 pandemic [10], yet few studies have evaluated the physical activity, dietary habits, and mental health of veterinary medical students affected by the COVID-19 pandemic. Additionally, while the previously aforementioned study evaluated the relationship between exercise with psychological well-being in veterinary students, that study was performed in a cohort that might have been inclined to less physical activity due to the season, and a study performed in a climate more likely to encourage outdoor activity has not been performed [9].

To date, little literature has evaluated the mental health of veterinary students affected by the COVID-19 pandemic after pandemic measures began to be lifted, and very few studies have evaluated the mental health of veterinary students impacted by the pandemic. Additionally, while one recent study has evaluated the relationship between physical activity and mental health in veterinary students [9], further studies in different cohorts of students are needed to support or refute the previous findings. Therefore, the main objective of this study was to describe physical activity, dietary habits, and mental health in a population of veterinary medical students at a public university in the United States after COVID-19 pandemic measures began to be lifted. A secondary objective was to examine factors associated with self-reported depression symptoms in this cohort of veterinary students.

## Materials and methods

### Research ethics statement

This study was approved by the university’s Institutional Review Board (IRB 202102605). The beginning of the survey instrument described the purpose of the study and that participation was voluntary, anonymous, and decision to participate had no impact on students’ academic standing. By approving the electronic consent to participate in the study, study participants were deemed to have consented to participate in the research prior to advancing to the survey questions.

### Study population

At time of the study, the University of Florida College of Veterinary Medicine (CVM) was home to 487 veterinary students. The general demographics of the veterinary student population was 82% female, 68% Caucasian, and a mean age at admission of 23 years (range, 19–45). At the time of this study, first-year, second-year, and third-year students were in didactic lectures and fourth-year students were in the last 6 months of their clinical rotations. Courses were offered in hybrid format, with lectures given in person while also providing livestreaming of lectures. Masking and physical distancing were no longer required at the time of this study, with mask requirements lifted on March 25, 2022 for the veterinary academic buildings and classrooms.

The University of Florida CVM provides on-site mental health services and wellness initiatives to students. The college currently has a wellness counselor on staff who is available at no cost to veterinary students for mental health support. The college, with assistance from the wellness counselor, also sponsors wellness initiatives, such as meditation and yoga, within the veterinary curriculum. In addition, the greater UF campus provides counseling for students through a number of programs, including the UF Counseling and Wellness Center, which provides short-term counseling at no cost, and the “U Matter, We Care” initiative.

### Study design

The investigation was designed as an observational cross-sectional study. An administrator within the CVM sent initial email invitations to all enrolled veterinary students at the CVM via class listserv emails. The email invitation contained a link to an online anonymous survey (Qualtrics, Seattle, WA). The first email invitation was sent March 28, 2022. The primary investigator (DL) sent two additional reminder emails on April 8 and April 25. The survey was closed on May 1, 2022. The study sample was a convenience, volunteer sample of veterinary students at the CVM. The beginning of the survey instrument described the purpose of the study and that participation was voluntary, anonymous, and decision to participate had no impact on students’ academic standing. It also contained an electronic consent to participate in the study, to which participants agreed prior to advancing to the survey questions. Respondents were informed that the data collected was anonymous and no information (name, student ID, etc.) that could identify individual participants was collected. IP addresses were monitored to prevent duplication of responses. Of the 167 total respondents, 55 were incomplete. Because this resulted in loss of key variables, these 55 respondents were removed from the study for analysis. Therefore, 112 students fully completed the survey and were included for analysis. Overall survey response rate was 23% (112/487).

### Survey instruments

The online survey consisted of 22 questions regarding demographics, dietary attitudes and habits, physical activity levels, depression, anxiety, and stress levels. Students were asked their age, gender identity, race, ethnicity, animal species focus, year of enrollment, and current estimated student loan debt (in US dollars). Students were also asked to quantify their study hours per week and class hours (including in-person and virtual lectures) per week.

Questions regarding students’ attitudes toward their health and nutrition were adapted from previous literature [11, 12]. Students were asked to rate their overall health (i.e., excellent, very good, good, average, poor, and very poor). Questions regarding nutrition were adapted from the validated 14-point Mediterranean Diet Survey [13]. A Mediterranean Diet adherence score was generated by counting the number of “yes” responses to the 14-point survey [13]. Physical activity questions were adapted from previous literature [11, 14, 15]. Students were asked to report how many hours per week they spent in total on vigorous physical activity (defined as “activities that get your heart racing and make you sweat (e.g., swimming, running, cycling at high speeds, cardio training, weight lifting, team sports)”) and on combined moderate (defined as “your heartbeat increases and you breathe faster (e.g., brisk walking, cycling, heavy gardening, running, or recreational sports)”) and vigorous physical activity. An additional question asked students to report their daily minutes performing yoga, meditation, prayer, or “other avenues to de-stress besides scholarly activity and exercise.”

Questions regarding students’ mental health were adapted from previous literature and included the American Psychiatric Association’s Diagnostic and Statistical Manual of Mental Disorders, fifth edition (DSM-5) Self-Rated Level 1 Cross-Cutting Symptom Measure survey, which has previously been evaluated as an assessment of college student mental health [16–18]. The DSM-5 symptom measure is comprised of 23 self-rated symptoms, which assess 13 mental health domains: depression, anger, mania, anxiety, somatic symptoms, suicidal ideation, psychosis, sleep disturbances, memory, repetitive thoughts and behaviors, dissociation, personality functioning, and substance use. The psychosis and substance use domains were removed for this study. Questions ask participants to rate how often they have been bothered by each symptom in the past two weeks, with response options of none (not at all), slight (rare, less than a day or two), mild (several days), moderate (more than half the days), severe (nearly every day). A score of mild or above in most domains (or slight or above for suicidal ideation) suggests a threshold for further investigation of clinically-relevant mental health concerns. The number of DSM-5 thresholds met by each participant was tallied. The prevalence of meeting the threshold for each of the aforementioned DSM-5 domains was determined for the total study population and by number of years in the veterinary program.

Additional questions regarding perceptions of stress level were adapted from previous literature [19]. Students were asked whether they felt stressed about time (always, sometimes, or never) [19]. Students were asked to rate from least stressful (1) to most stressful (5) concerns about the following aspects: education, research, work/life balance, family-related issues, personal health, relationships, organizational skills, time management, financial situation, living environment, physical environment of the CVM, and mental/emotional impact of the environment of the CVM. A free response question at the end of the survey invited students to provide additional comments if desired. Responses were included if students completed all survey items for the key variables.

### Statistical analysis

For the primary objective, categorical data were reported as observed frequencies, n (%), and continuous data were reported as mean ± SD and median (first, third quartiles). The distributions of the variables for age, hours spent studying per week, hours spent in class per week, current estimated student loan debt ($K), weekly hours of combined moderate or vigorous physical activity, weekly hours of vigorous activity, daily minutes of yoga, meditation, prayer or other de-stress techniques, or Mediterranean diet adherence score were compared between number of years in the veterinary program (1^st^, 2^nd^, 3^rd^, 4^th^ year) by using one-way analysis of variance (ANOVA) with Tukey’s post hoc test (normal), or the non-parametric Kruskal-Wallis followed by the Dunn’s test for multiple comparisons. Proportions of students by gender, race, DVM focus, feeling stressed about time, self-reported health rating, and mental health domain were compared between number of years in the veterinary program by using a Chi-square test.

For the secondary objective, the association between depression symptoms and investigated exposure factors in veterinary students was examined using logistic regression analysis. The primary outcome of interest was depression symptoms, which was defined as having a response value of “moderate” or “severe” for any DSM-5 depression domain question. Physical activity, stress, student debt, diet, gender identity, age, race, and number of years in veterinary program were investigated as exposure factors associated with depression. The analysis included 42 veterinary students who met the above definition of depression symptoms, and 17 who did not (reported a score of “none” for all mental health domains, including depression). In the univariable analysis, variables with values of p ≤ 0.25 were further examined using multivariable logistic regression analysis [20]. Association between variables was examined (i.e., those with p ≤ 0.15), and when a pair of variables was associated by use of a chi-squared test (two-tailed), the exposure variable judged as most biologically plausible was further examined in the analysis. Confounding effects of gender or number of years in the veterinary program on investigated associations between race, student loan, vigorous exercise, or feeling stressed about time and depression were measured by assessing changes (i.e., > 20%) in calculated crude odds ratios and adjusted odd ratios. Goodness of fit of the model was explored by the Hosmer-Lemeshow goodness-of-fit chi-squared statistic.

## Results

Of 112 students who completed the survey, 88 (78%) were females, 87 (78%) were Caucasian, and the median student age was 26 years (Table 1). The demographic makeup was similar to the overall veterinary school demographics.

**Table 1:** Demographics by number of years in veterinary program.

Variable	All	Number of years in veterinary program				
		1^st^ year	2^nd^ year	3^rd^ year	4^th^ year	p-value
	n = 112	n = 30	n = 20	n = 41	n = 21	
**Gender**						
*Female*	88 (78)	26 (87)	12 (60)	32 (78)	18 (86)	0.40
*Male*	19 (17)	3 (10)	6 (30)	7 (17)	3 (14)	
*Gender non-binary*	2 (2)	1 (3.33)	1 (5)	0 (0)	0 (0)	
*Prefer not to disclose*	3 (3)	0 (0)	1 (5)	2 (5)	0 (0)	
**Age**	27 ± 4	25 ± 3	27 ± 6	26 ± 2	28 ± 3	<0.01
	26 (24, 27)	24 (23, 26)^a^	25 (24, 26)^ab^	26 (25, 27)^b^	27 (26, 29)^b^	
**Race/ethnicity**						0.23
*African-American*	0	0	0	0	0	
*American-Indian*	0	0	0	0	0	
*Asian*	2 (2)	1 (3)	0	1 (2)	0	
*Caucasian*	87 (78)	23 (77)	13 (65)	33 (80)	18 (86)	
*Hispanic/Latino*	14 (12)	5 (17)	4 (20)	3 (7)	2 (10)	
*Hawaiian/Pacific Islander*	0	0	0	0	0	
*Other*	5 (4)	1 (3)	0	3 (7)	1 (5)	
*Prefer not to disclose*	4 (4)	0	3 (15)	1 (2)	0	
**DVM focus**						0.68
*Small animal*	59 (53)	17 (57)	10 (50)	19 (46)	13 (62)	
*Equine*	8 (7)	1 (3)	2 (10)	5 (12)	0	
*Large animal*	7 (6)	1 (3)	2 (10)	4 (10)	0	
*Farm animal*	1 (1)	0	0	0	1 (5)	
*Mixed*	15 (13)	4 (13)	3 (15)	4 (10)	4 (19)	
*Exotics/Wildlife*	18 (16)	6 (20)	2 (10)	8 (20)	2 (10)	
*Other*	4 (4)	1 (3)	1 (5)	1 (2)	1 (5)	

Data are reported as observed frequencies, n (%) or as mean ± SD and median (first, third quartiles).

^ab^ DVM student groups with different superscripts are different from each other (p < 0.05).

### Physical activity, diet, and self-reported stress by number of years in the veterinary program

Number of hours spent studying per week were higher in second year (median 40 hours) than in third (25 hours) or fourth year (14 hours) (p < 0.01; Table 2). Median current student loan burden varied from $47,000 to $178,000 and loan amounts were higher in fourth year students (median $178,000) as compared to 1^st^ year students ($47,000; p = 0.01).

**Table 2:** Weekly activity, student debt, and self-reported stress and overall health.

Variable	All	Number of years in veterinary program				
		1^st^ year	2^nd^ year	3^rd^ year	4^th^ year	p-value
	n = 112	n = 30	n = 20	n = 41	n = 21	
**Hours spent studying per week**	31 ± 26	28 ± 14	52 ± 43	27 ± 16	26 ± 24	<0.01
	30 (15, 40)	29 (20, 40)^ab^	40 (25, 50)^a^	25 (15, 30)^b^	14 (10, 40)^b^	
**Hours spent in class per week**	33 ± 35	28 ± 13	41 ± 34	26 ± 18	48 ± 70	0.30
	25 (15, 40)	25 (20, 35)	30 (19, 49)	21 (15, 29)	22 (0, 60)	
**Student debt ($K)**	92 ± 77	73 ± 81	109 ± 54	76 ± 67	134 ± 89	0.01
	80 (28, 150)	47 (0, 100)^a^	95 (75, 155)^ab^	80 (0, 120)^ab^	178 (80, 199)^b^	

**Do you feel stressed about time?**						0.05
*Always*	59 (53)	17 (57)	14 (70)	15 (37)	13 (62)	
*Sometimes*	46 (41)	12 (40)	6 (30)	20 (49)	8 (38)	
*No*	7 (6)	1 (3)	0	6 (15)	0	
**Do you feel stressed about time?**						0.06
*Always*	59 (53)	17 (57)	14 (70)	15 (37)	13 (62)	
*Sometimes/Never*	53 (47)	13 (43)	6 (30)	26 (63)	8 (38)	

**Self-reported overall health rating**						0.14
*Excellent–very good*	35 (31)	11 (37)	6 (30)	15 (37)	3 (14)	
*Good–average*	66 (59)	17 (57)	10 (50)	25 (61)	14 (67)	
*Poor–very poor*	11 (10)	2 (7)	4 (20)	1 (2)	4 (19)	

Data are reported as observed frequencies, n (%) or as mean ± SD and median (first, third quartiles).

^ab^ DVM student groups with different superscripts are different from each other (p < 0.05).

Weekly hours of vigorous physical activity were lower in fourth year veterinary students (median 0.5 hours) compared to first (3 hours) and third year students (3 hours) (p <0.01; Table 3). There was no difference in Mediterranean diet adherence score between number of years in the veterinary program (p = 0.91). S1 Table shows frequency of responses to 14-Point Mediterranean Diet Survey and additional nutrition questions.

**Table 3:** Physical activity and diet by number of years in veterinary program.

Variable	All	Number of years in veterinary program				
		1^st^ year	2^nd^ year	3^rd^ year	4^th^ year	p-value
	n = 112	n = 30	n = 20	n = 41	n = 21	
**Weekly hours of combined moderate + vigorous physical activity**	4 ± 5	4 ± 3	3 ± 2	4 ± 3	5 ± 11	0.03*
	3 (2, 5)	4 (2, 7)	3 (1.5, 3.25)	3 (2, 5)	2 (1.5, 3)	
**Weekly hours of vigorous physical activity**	3 ± 5	3 ± 4	2 ± 2	4 ± 3	3 ± 11	<0.01
	2 (0, 5)	3 (0, 5)^b^	2 (0, 3.5)^ab^	3 (1,6)^b^	0.5 (0, 1.5)^a^	
**Daily minutes of yoga, meditation, prayer or other destress**	32 ± 47	31 ± 38	27 ± 38	45 ± 62	11 ± 16	0.12
	10 (0, 60)	13 (2, 40)	10 (0, 60)	30 (0, 60)	5 (0, 20)	
**Mediterranean diet adherence score**	7 ± 2	7 ± 2	7 ± 2	7 ± 2	7 ± 2	0.91
	7 (6, 9)	7 (6, 9)	8 (6, 9)	7 (6, 8)	7 (5, 9)	

Data are reported as mean ± SD and median (first, third quartiles).

^ab^ DVM student groups with different superscripts are different from each other (p < 0.05).

*No difference between groups on post-hoc multiple comparisons analysis

Table 4 reports median scores for perception of stress regarding aspects of students’ education and lives. Highest median scores were seen for concerns about education, work/life balance, and financial situation.

**Table 4:** Perceptions of stress among 112 veterinary students.

	Score
**Concerns about my education**	4 (3, 5)
**Concerns about my research**	1.5 (1, 4)
**Work/life balance**	4 (3, 5)
**Family-related issues**	2 (1, 4)
**Personal health**	3 (2, 4)
**Relationships**	3 (2, 4)
**Organizational skills**	2 (1, 3)
**Time management**	3 (2, 4)
**Financial situation**	4 (3, 5)
**Living environment**	2 (1, 3)
**Physical environment of the CVM**	2 (1, 3)
**Mental and emotional impact of the environment of the CVM**	3 (2, 5)

Note: items were scored on a 5-point scale (1 = least stressful, 5 = most stressful). Data presented as median and interquartile range.

### Mental health by number of years in the veterinary program

The frequency of students who met the threshold for further investigation of clinically-relevant depression symptoms was high and varied from 46% (19/41) in third year students to 71% (15/21) in fourth year students; but the difference was not significant (p = 0.29) (Table 4). The frequency of students who met the threshold for suicidal ideation symptoms was higher among fourth year students (29%; 6/21) than first (7%; 2/30) and third year students (7%; 3/41), *p* = 0.04). Seventeen (14%) of students indicated no DSM-5 mental health concerns (Table 5).

**Table 5:** Frequency of DSM-5 domain concerns in 112 veterinary students.

DSM-5 mental health domain	All	Number of years in veterinary program				
		1^st^ year	2^nd^ year	3^rd^ year	4^th^ year	p-value
	n = 112	n = 30	n = 20	n = 41	n = 21	
I Depression	63 (56)	17 (57)	12 (60)	19 (46)	15 (71)	0.29
II Anger	59 (53)	14 (47)	11 (55)	18 (44)	16 (76)	0.09
III Mania	44 (39)	12 (40)	9 (45)	16 (39)	7 (33)	0.90
IV Anxiety	75 (67)	22 (73)	13 (65)	23 (56)	17 (81)	0.20
V Somatic Symptoms	56 (50)	17 (57)	8 (40)	17 (41)	14 (67)	0.18
VI Suicidal Ideation	16 (14)	2 (7)^a^	5 (25)^ab^	3 (7)^a^	6 (29)^b^	0.04
VII Sleep disturbances	55 (49)	19 (63)	9 (45)	16 (39)	11 (52)	0.23
VIII Memory	32 (29)	7 (23)	7 (37)	9 (22)	9 (43)	0.26
IX Repetitive thoughts and behaviors	30 (27)	6 (20)	7 (35)	10 (24)	7 (33)	0.58
X Dissociation	37 (33)	10 (33)	7 (35)	11 (27)	9 (43)	0.65
XI Personality Functioning	47 (42)	11 (37)	10 (50)	15 (37)	11 (52)	0.51
Number of DSM-5 domain threshold concerns	5 ± 3	5 ± 3	5 ± 4	4 ± 3	6 ± 3	0.12
	4 (2, 7)	4 (3, 6)	4.5 (0, 9)	3 (1, 6)	6 (3, 9)	

Data are reported as observed frequencies, n (%) or as mean ± SD and median (first, third quartiles).

^ab^ DVM student groups with different superscripts are different from each other (p < 0.05).

The depression domain was associated (p < 0.05) with all other DSM-5 mental health domains; therefore, the remainder of the analyses focused on depression as the outcome variable.

### Exposure factors associated with depression symptoms

Using univariable logistic regression, the variables for student debt, weekly hours of vigorous physical activity, feeling stressed about time, and self-reported overall health rating had values of p < 0.25 and were further examined (S2 Table).

In the multivariable analysis, the odds of self-reported depression symptoms were 7.6 times lower in students engaged in high levels of vigorous exercise, compared to those with low levels, after controlling for number of years in the veterinary program (adjusted OR = 0.13; 95% CI = 0.02, 0.77; p = 0.02) (S3 Table). The Hosmer-Lemeshow goodness of fit test (1.88; df = 4; p = 0.75) indicated that there is no evidence of a poor fit for the data. In the final model, the crude odds ratio for self-reported depression symptoms (0.13) did not change in students engaged in high levels of vigorous exercise, when the variable for number of years in the veterinary program was added to the model, suggesting that the observed association between vigorous exercise and depression symptoms was not confounded by number of years in the veterinary program.

The odds of self-reported depression symptoms were 68.7 times higher in students who always felt stress about time, compared to those sometimes or never felt stressed, after controlling for number of years in the veterinary program (adjusted OR = 68.70; 95% CI = 7.63, 618.4; p < 0.01). The Hosmer-Lemeshow goodness of fit test (8.1; df = 4; p = 0.08) indicated that there is no evidence of a poor fit of the data. In the final model, the odds ratio for self-reported depression symptoms changed from 34.5 (crude odds ratio) to 68.7 (adjusted odds ratio) (99% change) in students who always felt stress about time, when the variable for number of years in the veterinary program was added to the model, suggesting that years in the veterinary program had a negative confounding effect on the association between stress and depression symptoms.

## Discussion

This study reports novel information regarding the burden of mental health concerns in veterinary students directly affected by the COVID-19 pandemic, after pandemic measures began to be lifted. This study also identified an association between physical activity and self-reported depression symptoms, although a causal link could not be made. Importantly, this study confirms similar associations as seen in a previous single university study, but in a different cohort, region, and timepoint [9].

A high frequency of mental health concerns was reported by the students in this study. The high frequency of mental health concerns despite provision of counselling services to the students in this population strengthens the finding that the frequency of mental health concerns remained high and that more needs to be done to address the mental health concerns of veterinary students. The presence of mental health providers at the university is meant to support and treat the students who need assistance, and whether it does or does not reduce the frequency of mental health concerns cannot be deduced from this study, or from any other published study to the authors’ knowledge. Due to the cross-sectional study design, a direct causal effect of the COVID-19 pandemic on mental health cannot be made. However, previous literature has demonstrated an effect of COVID-19 pandemic on students’ mental health and it is possible that the high frequency of mental health concerns seen in this study population is related to residual effects of the pandemic, despite the fact that daily student life had resumed all pre-pandemic activities and expectations at the time of this study [4, 21]. A recent study found that depression increased and physical activity decreased among US college students during the 2020 COVID-19 stay-at-home order [21]. In that study, freshman and white students were more likely to report increased symptoms of depression. The majority of participants in our study were Caucasian, and due to the low level of minority students included in this study, further investigation of this factor could not be pursued in the current study.

While not statistically significant across all mental health domains, there did appear to be a higher burden of self-reported mental health symptoms in 2^nd^ and 4^th^ year DVM students. The second year veterinary students in this study would have started the veterinary program during the pandemic (fall 2020), with many classes being virtual at the start of their program. This may have affected these students’ ability to develop camaraderie and build connections with other students, which may have impacted students’ wellbeing. Additionally, the third and fourth year students would have experienced the veterinary program both pre- and post- pandemic closures, and the impact of this experience on their wellbeing could not be evaluated in the current study. The fourth year students, in particular, might have had the most disruption to their academic career from the pandemic, experiencing the transition to virtual classes in their second year and then experiencing disruption of their clinical rotations at the beginning of their third year. The detrimental effects of the COVID-19 pandemic on veterinarian mental health were recently shown in a large-scale study of veterinarians, and it is possible that similar effects were seen in this student cohort [3].

In this study, the odds of meeting the threshold for further investigation of clinically-relevant depression were 7.6 times lower in students engaged in high levels of vigorous exercise, compared to those with low levels, after controlling for number of years in the veterinary program. Previous studies have supported a similar link between physical activity, wellbeing, and mental health [22–25]. A recent study found a similar association between exercise and psychological well-being in veterinary students, although that study was performed prior to the COVID-19 pandemic [9]. Interestingly, the amount of weekly exercise in our cohort of students was reportedly higher than in the previous study (mean of 4 hours versus mean of 2 exercise sessions of 34 minutes each in the previous study) [9]. Our study was performed in Florida during spring, a time of year that often encourages outdoor activity, while the previous study was performed in Kansas in the fall, during a time of year where perhaps less outdoor activity is possible. Additionally, it is possible that the provision of hybrid courses, something instituted after the pandemic, allowed students in our study more freedom to pursue exercise and activities outside of the curriculum. Despite the higher level of physical activity in our students, there were still high rates of mental health concerns, ascertaining that the link between mental health and physical activity is likely more complex than either study could evaluate. This study, as well as the previous study, does not prove causality or directionality. It is possible that students who exercised more had better mental health, but it is also possible that students with better mental health were more likely to exercise. When compared to the general US population, the veterinary students in this study appeared to be more active than the general US population. Median weekly hours of vigorous exercise among students in this study was two hours, and approximately 60% of students reported weekly vigorous physical activity above the US physical activity guidelines (>75 minutes per week), as compared to about 20% of the general US population [26].

While stress was a self-reported variable in this study, it was consistently found to be associated with depression symptoms, and students reported the highest stress scores for concerns regarding their education, work/life balance, or financial situation. Similarly, a previous study of stressors of veterinary students in New Zealand identified workload (i.e., classroom contact time and study time), grades, and finances as significant risk factors for stress [27]. A more recent study found that heavy academic workload, being behind in studies, inefficient studying, and concerns about academic performance were the most commonly identified stressors among a cohort of veterinary students [9]. In a study of veterinary students during the COVID-19 pandemic, sleeping difficulties, stress regarding confine difficulty sleeping, stress associated with confinement, and family conflicts were identified as risk factors for anxiety among veterinary students [4]. Because student stress was associated with mental health concerns in this study, reducing student stress through stress relief programs or teaching students effective coping strategies for stress might be important targets for improving veterinary student wellbeing.

This study had several limitations. This study only included veterinary students at the University of Florida and the external validity of this study therefore could be a limitation. However, this limitation affects the majority of studies evaluating veterinary student mental health, as these are usually limited to single institution studies [4, 6, 8, 9]. The mental health of students in Florida in April 2022 may not be representative of student experiences at other veterinary schools, or students who did not attend school during the COVID-19 pandemic, and a larger, national as well as international study might find different results. A recent study of the mental wellbeing of pharmacy students across 14 countries determined that more than a third of students had low mental wellbeing [28]. In that study, a variety of factors were associated with low mental wellbeing, including academic distress, gender, physical activity, and body weight. The results of numerous studies, including our study, support that the mental health of professional students across many countries is a serious point of concern and one that school administrators should strive to address [6, 9, 10, 19, 27, 28]. Additionally, the APA DSM-5 survey suggests a threshold for further investigation of clinically-relevant mental health concerns, but does not definitively diagnose these mental health concerns. However, this measure is often used clinically and has previously been evaluated as an assessment of college student mental health and this measure is used by the wellness counselor at our institution to assess veterinary students for further investigation [16, 17]. This measure was also recently validated and found to produce internally consistent results [18].

Voluntary participation in the survey as well as low survey response rate may have led to selection bias. Students experiencing poor mental health may have been more likely to complete the survey, or alternatively, this study may have failed to represent students with the poorest mental health, who may not have had the time or energy to complete the survey. Regardless of whether the estimated prevalence was under- or over-estimated, the prevalence was high, justifying additional risk management measures among veterinary students. Although the survey response rate was low in this study (23%), a previous study of the reliability of low survey response rates found that a response rate of 20–25% in a study with a sample size of <500 provided fairly confident estimates of the population [29]. All survey data was self-reported, but the survey was anonymous to reduce social desirability bias. An inherent limitation was the use of a cross-sectional study design and therefore a temporal and causal relationship between variables could not be established. A longitudinal study might have been better able to detect the impact of the COVID-19 pandemic in this cohort of students. Many veterinary schools now provide mental health support services and wellness initiatives to their veterinary students, but students’ access to these services may have been impacted by the COVID-19 pandemic [30]. Additional limitations include the small sample size, particularly of certain sub-groups, and the dichotomization of variables such as gender identity and racial identity. Because sample size was small, assessment of interaction between predictor variables (e.g., vigorous exercise and student debt) could not be performed [6, 9, 10, 19, 27, 28].

The frequency of veterinary students affected with self-reported depression symptoms and other mental health concerns in this study was high. Self-reported stress and low weekly vigorous physical activity were associated with depression symptoms. With mental health concerns in veterinary students found to be a serious factor in this study and previous studies, effective strategies to improve the mental health and overall wellbeing of veterinary students must be developed, and further longitudinal studies should be pursued to evaluate the residual effect of the COVID-19 pandemic on students’ mental health over time.

## Supporting information

S1 Table Survey responses of 112 veterinary students to the fourteen-point mediterranean diet survey. (DOCX)

S2 Table Univariate logistic regression for prediction of moderate-severe depression symptoms in 59 veterinary students. (DOCX)

S3 Table Multivariate logistic regression for prediction of moderate-severe depression symptoms in 59 veterinary students. (DOCX)

S1 File (PDF)

S1 Data (XLSX)
