# Supplementary material for: Cross-sectional study of physical activity, dietary habits, and mental health of veterinary students after lifting of COVID-19 pandemic measures
Source: PLoS One. 2023 Sep 14;18(9):e0291590. doi: 10.1371/journal.pone.0291590 (PMC10501662; doi:10.1371/journal.pone.0291590)
Supplement: S1 Table — (DOCX) [file pone.0291590.s001.docx]

**Supplemental Table 1**. Survey responses of 112 veterinary students to the Fourteen-Point Mediterranean Diet Survey

| **Question** | **N (Yes)** | **%** |
| --- | --- | --- |
| Do you eat products such as milk or cheese? | 103 | 92 |
| Do you use olive oil as your main culinary fat? | 86 | 77 |
| On a given day, do you consume ≥ 4 tablespoons of olive oil (including oil used for frying, salads, out-of-house meals, etc)? | 23 | 21 |
| Do you consume ≥ 2 vegetable servings (≥ 1 serving raw or as a salad) per day (1 serving = 200 g [consider dishes as half a serving])? | 74 | 66 |
| Do you consume ≥ 3 fruit units (including natural fruit juices) per day? | 55 | 49 |
| Do you consume ≥ 7 glasses of wine per week? | 9 | 8 |
| Do you consume ≥ 3 servings of legumes per week (1 serving = 150 g)? | 50 | 45 |
| Do you consume ≥ 3 servings of fish or shellfish per week (1 serving = 100-150 g of fish or 4-5 units of 200 g of shellfish)? | 18 | 16 |
| Do you consume ≥ 3 servings of nuts (including peanuts) per week (1 serving = 30 g)? | 53 | 47 |
| Do you prefer to eat chicken, turkey, or rabbit meat over veal, pork, hamburger, or sausage? | 79 | 71 |
| Do you consume ≥ 2 servings of vegetables, pasta, rice, or other dishes seasoned with sofrito (sauce made with tomato and onion, leek, or garlic and simmered with olive oil) per week? | 58 | 52 |
| Do you have <1 serving of red meat, hamburger, or meat products (ham, sausage, etc) per day (1 serving = 100-150 g)? | 88 | 79 |
| Do you consume <1 serving of butter, margarine or cream per day (1 serving = 12 g)? | 77 | 64 |
| How many sweet or carbonated beverages do you consume per day?  *<1*  *2*  *3*  *≥ 4* | 101  11  5  3 | 90  10  4  2.7 |
