# Supplementary material for: Cross-sectional study of physical activity, dietary habits, and mental health of veterinary students after lifting of COVID-19 pandemic measures
Source: PLoS One. 2023 Sep 14;18(9):e0291590. doi: 10.1371/journal.pone.0291590 (PMC10501662; doi:10.1371/journal.pone.0291590)
Supplement: S2 Table — (DOCX) [file pone.0291590.s002.docx]

**Supplemental Table 2**. Univariate Logistic Regression for Prediction of Moderate-Severe Depression Symptoms in 59 Veterinary Students

| **Variable** | **Category** | **Depression No**  **n = 17** | **Depression**  **Yes n = 42** | **OR** | **95% CI** | **p** |
| --- | --- | --- | --- | --- | --- | --- |
| Gender | Female Male | 12 4 | 31 11 | 1.00 1.11 | Referent 0.29, 4.31 | NA 0.88 |
| Age (years) | 22 to 26 27 to 46 | 11 6 | 26 16 | 1.00 1.13 | Referent 0.35, 3.65 | NA 0.84 |
| Race | Caucasian Other | 13 4 | 31 11 | 1.00 1.15 | Referent 0.31, 4.3 | NA 0.83 |
| Number of years in the veterinary program | 1  2  3  4 | 3 6 7 1 | 9 9 13 11 | 1.00 0.50 0.62 3.66 | Referent 0.09, 2.64 0.13, 3.06 0.32, 41.59 | NA 0.41 0.56 0.29 |
| Weekly hours of vigorous exercise | Low: ≤ 0.06 h/week Medium: 0.07 – 3 h/week  High: 3+ h/week | 2  7  8 | 18  15  9 | 1.00  0.24  0.13 | Referent  0.04, 1.32  0.02, 0.71 | NA  0.10  0.02 |
| Student debt ($) | 0 to 99k 100k to 300k | 10 7 | 16 26 | 1.00 2.32 | Referent 0.74, 7.33 | NA 0.15 |
| Self-reported overall health rating | Excellent – Good  Average – Very Poor | 16  1 | 18  24 | 1.00  21.33 | Referent  2.58, 176.08 | NA  <0.01 |
| Do you feel stressed about time? | Never/Sometimes Always | 14 3 | 5 37 | 1.00 34.53 | Referent 7.27, 163.99 | NA < 0.01 |

OR = odds ratio; 95%CI: 95% confidence interval
