# Supplementary material for: Cross-sectional study of physical activity, dietary habits, and mental health of veterinary students after lifting of COVID-19 pandemic measures
Source: PLoS One. 2023 Sep 14;18(9):e0291590. doi: 10.1371/journal.pone.0291590 (PMC10501662; doi:10.1371/journal.pone.0291590)
Supplement: S3 Table — (DOCX) [file pone.0291590.s003.docx]

**Supplemental Table 3**. Multivariate Logistic Regression for Prediction of Moderate-Severe Depression Symptoms in 59 Veterinary Students

| **Variable** | **Category** | **N** | **Crude OR** | **95% CI** | **p** | **Adjusted OR** | **95% CI** | **p** |
| --- | --- | --- | --- | --- | --- | --- | --- | --- |
| Model 1 | | | | | | | | |
| Weekly hours of vigorous exercise  DVM year | Low Medium  High  1  2  3  4 | 20  22  17  12 15 20 12 | 1.00 0.24 0.13  1.00 0.50 0.62 3.66 | Reference 0.04, 1.32 0.02, 0.71  Reference 0.09, 2.64 0.13, 3.06 0.32, 41.59 | NA 0.10 0.02  NA 0.41 0.56, 0.29 | - 0.24  0.13  - 0.45 0.75 3.46 | -  0.04, 1.41  0.02, 0.77   0.07, 2.76 0.14, 4.09 0.28, 43.05 | - 0.11  0.02  - 0.38 0.73 0.33 |
| Model 2 | | | | | | | | |
| Do you feel stressed about time?   DVM year | Never/Sometimes Always  1  2  3  4 | 19 40  12 15 20 12 | 1.00 34.53  1.00  0.50  0.62  3.66 | Reference  7.27, 163.99  Reference  0.09, 2.64  0.13, 3.06  0.32, 41.59 | NA  < 0.01  NA  0.41  0.56,  0.29 | - 68.70  - 0.13 0.81 2.77 | - 7.63, 618.4  - 0.01, 2.01 0.08, 8.03 0.12, 63.68 | - < 0.01  - 0.14 0.85 0.52 |
