# Supplementary material for: Cross-sectional study of physical activity, dietary habits, and mental health of veterinary students after lifting of COVID-19 pandemic measures
Source: PLoS One. 2023 Sep 14;18(9):e0291590. doi: 10.1371/journal.pone.0291590 (PMC10501662; doi:10.1371/journal.pone.0291590)
Supplement: S1 File — (PDF) [file pone.0291590.s004.pdf]

Default Question Block

Thank you for your interest in participating in this survey (IRB study#202102605).

This research study will describe the physical activity, nutrition, and wellness of veterinary professional and graduate students at the University of Florida CVM.

The survey will take approximately 15 minutes and you will answer questions about your physical activity, diet, and markers of wellbeing.

Your participation is completely voluntary, anonymous, and your decision to participate or not participate will have no impact on your academic standing. You can skip any questions you are not comfortable with and stop at any point. There are no direct benefits for your participation. The information gained from this study will be used to develop program initiatives to promote exercise and healthy eating habits. There are no foreseeable risks to your participation. To protect your confidentiality, no identifiers will be collected. This survey was also approved through the University of Florida IRB.

If you have any questions about this research, please contact the PI Dr. Daniela Luethy at dluethy@ufl.edu or 352-294-5177. For questions regarding your rights as a research participant, please contact the IRB-02 office at 352-392-0433

We appreciate your time and effort for this research study. If you agree to participate in this study and complete the survey, please click "I agree" below.

○ I agree

Overall, how would you rate your health?

- Excellent
- Very good
- Good
- Average
- Poor
- Very poor

On average over the past 4 weeks, how many hours per week did you spend studying?

On average over the past 4 weeks, how many hours per week did you spend in class (attending lectures or viewing online lectures, in teaching laboratories)?

Do you feel stressed about time?

- Yes, all the time
- Sometimes
- No, I have time to do what I need to do

The questions below ask about your physical activity.

In an average week, how many HOURS PER WEEK do you spend in total on moderate and vigorous physical activities where your heartbeat increases and you breathe faster (e.g. brisk walking, cycling as a means of transport or as exercise, heavy gardening, running or recreational sports)?

In an average week, how many HOURS PER WEEK do you spend in total on vigorous

physical activities (this includes activities that get your heart racing and make you sweat (e.g. swimming, running, cycling at high speeds, cardio training, weight-lifting, team sports)?

In an average day, how many MINUTES PER DAY do you meditate/do yoga/pray/or have other avenues to destress besides scholarly activity and exercise?

In what forms of physical activity or exercise do you participate (check all that apply):

- ☐ a. Walking/Hiking
- ☐ b. Jogging/Running
- ☐ c. Cycling
- ☐ d. Yoga/Pilates
- ☐ e. Team sports (basketball, soccer, football, rugby, hockey, etc.)
- ☐ f. HIIT
- ☐ g. Tennis/Raquetball/Squash
- ☐ h. Golf
- ☐ i. Martial arts
- ☐ j. Swimming
- ☐ k. Horseback riding
- ☐ l. Other:

The following questions will ask you about your dietary habits.

	Yes	No
Do you eat products such as milk or cheese?	○	○
Do you use olive oil as your main culinary fat?	○	○
On a given day, do you consume ≥ 4 tablespoons of olive oil (including oil used for frying, salads, out-of-house meals, etc)?	○	○
Do you consume ≥ 2 vegetable servings (≥ 1 serving raw or as a salad) per day (1 serving = 200 g [consider dishes as half a serving])?	○	○
Do you consume ≥ 3 fruit units (including natural fruit juices) per day?	○	○
Do you consume ≥ 7 glasses of wine per week?	○	○
Do you consume ≥ 3 servings of legumes per week (1 serving = 150 g)?	○	○
Do you consume ≥ 3 servings of fish or shellfish per week (1 serving = 100-150 g of fish or 4-5 units of 200 g of shellfish)?	○	○
Do you consume ≥ 3 servings of nuts (including peanuts) per week (1 serving = 30 g)?	○	○
Do you prefer to eat chicken, turkey, or rabbit meat over veal, pork, hamburger, or sausage?	○	○
Do you consume ≥ 2 servings of vegetables, pasta, rice, or other dishes seasoned with sofrito (sauce made with tomato and onion, leek, or garlic and simmered with olive oil) per week?	○	○
Do you have <1 serving of red meat, hamburger, or meat products (ham, sausage, etc.) per day (1 serving = 100-150 g)?	○	○
Do you consume <1 serving of butter, margarine, or cream per day (1 serving = 12 g)?	○	○

How many sweet or carbonated beverages do you consume per day?

- ○ Less than or equal to 1
- ○ 2
- ○ 3
- ○ Greater than or equal to 4

On a weekly basis, what percentage of your diet is composed of a personally cooked meal that is plant-based?

- Less than 5%
- 6-10%
- 10-30%
- 31-50%
- >50%

How important is nutrition in your daily life?

- Very important
- Somewhat important
- Neutral
- Not important
- Important, but I don't have time to focus on it right now

Since beginning your program, how has your weight changed?

- Gained <5 pounds
- Gained 5-10 pounds
- Gained 10-20 pounds
- Gained >20 pounds
- Lost <5 pounds
- Lost 5-10 pounds
- Lost 10-20 pounds
- Lost >20 pounds
- Weight has not changed

The questions below ask about things that might have bothered you. For each question, circle the number that best describes how much (or how often) you have been bothered by each problem during the past TWO (2) WEEKS.

During the past TWO (2) WEEKS, how much (or how often) have you been bothered by the following problems?

	None (not at all)	Slight (rare, less than a day or two)	Mild (several days)	Moderate (more than half the days)	Severe (nearly every day)
1. Little interest or pleasure in doing things?	○	○	○	○	○
2. Feeling down, depressed, or hopeless?	○	○	○	○	○
3. Feeling more irritated, grouchy, or angry than usual?	○	○	○	○	○
4. Sleeping less than usual, but still have a lot of energy?	○	○	○	○	○
5. Starting lots more projects than usual or doing more risky things than usual?	○	○	○	○	○
6. Feeling nervous, anxious, frightened, worried, or on edge?	○	○	○	○	○
7. Feeling panic or being frightened?	○	○	○	○	○
8. Avoiding situations that make you anxious?	○	○	○	○	○
9. Unexplained aches and pains (e.g., head, back, joints, abdomen, legs)?	○	○	○	○	○
10. Feeling that your illnesses are not being taken seriously enough?	○	○	○	○	○
11. Thoughts of actually hurting yourself?	○	○	○	○	○

12. Feeling that someone could hear your thoughts, or that you could hear what another person was thinking?	○	○	○	○	○
13. Problems with sleep that affected your sleep quality over all?	○	○	○	○	○
14. Problems with memory (e.g., learning new information) or with location (e.g., finding your way home)?	○	○	○	○	○
15. Unpleasant thoughts, urges, or images that repeatedly enter your mind?	○	○	○	○	○
16. Feeling driven to perform certain behaviors or mental acts over and over again?	○	○	○	○	○
17. Feeling detached or distant from yourself, your body, your physical surroundings, or your memories?	○	○	○	○	○
18. Not knowing who you really are or what you want out of life?	○	○	○	○	○
19. Not feeling close to other people or enjoying your relationships with them?	○	○	○	○	○

From least stressful (1) to most stressful (5), please rate the following with regards to your life as a student at the University of Florida College of Veterinary Medicine:

	1	2	3	4	5
Concerns about my education					
Concerns about my research					
Work/life balance					
Family-related issues					
Personal health					
Relationships					
Organizational skills					
Time management					
Financial situation					
Living environment					
Physical environment of the CVM					
Mental and emotional impact of the environment of the CVM					

What is your age in years?

What is your gender?

- Female
- Male
- Non-binary / third gender
- Prefer not to say

What is your racial/ethnic background?

- African-American
- American Indian or Alaska Native
- Asian
- Caucasian
- Hispanic or Latino
- Native Hawaiian or Pacific Islander
- Other
- Prefer not to say

In which degree program are you enrolled?

- DVM
- DVM/MPH
- Master's
- PhD
- Other: ____________

What year did you begin your veterinary or graduate program?

- 2022
- 2021
- 2020
- 2019
- 2018
- 2017
- 2016
- 2015
- Prior to 2015

If you are in a DVM program, what is your species focus/interest?

- Small animal
- Equine
- Large animal
- Food animal
- Mixed animal
- Exotics/wildlife
- Other ______
- Not in a DVM program

What is your current estimated student loan debt amount in US dollars?

______

Please feel free to make any comments you would like to make about your current physical activity, diet, or wellbeing, or any comments on this survey. Thank you for your time and participation in this study.

______

©University of Florida
Gainesville, FL 32611
Terms of Use

Powered by Qualtrics
